# Robot-assisted pedicle screw insertion versus navigation-based and freehand techniques for posterior spinal fusion in scoliosis: a systematic review and meta-analysis

**DOI:** 10.1007/s43390-024-00879-y

**Published:** 2024-04-15

**Authors:** Abdulrahman O. Al-Naseem, Abdullah Al-Muhannadi, Mohammad Ramadhan, Alwaleed Alfadhli, Yousef Marwan, Roozbeh Shafafy, Muhammad M. Abd-El-Barr

**Affiliations:** 1 Jaber Al-Ahmad Hospital, Ministry of Health, Kuwait City, Kuwait; 2 School of Medicine, University of Manchester, Manchester, UK; 3 School of Medicine, Royal College of Surgeons, Dublin, Ireland; 4 Department of Surgery, College of Medicine, Health Sciences Centre, Kuwait University, Kuwait City, Kuwait; 5 Division of Surgery & Interventional Science, University College London, London, UK; 6 Department of Spinal Surgery, Royal National Orthopaedic Hospital NHS Foundation Trust, Stanmore, UK; 7 Department of Neurosurgery, Division of Spine, Duke University Medical Centre, Durham, USA

**Keywords:** Spine, Scoliosis, Robot, Navigation, Freehand, Fusion

## Abstract

**Purpose:**

The role of robotics in spine surgery remains controversial, especially for scoliosis correction surgery. This study aims to assess the safety and efficacy of robotic-assisted (RA) surgery specifically for scoliosis surgery by comparing RA to both navigation systems (NS) and conventional freehand techniques (CF).

**Methods:**

As per the Preferred Reporting Items for Systematic Reviews and Meta-analyses (PRISMA) guidelines, a systematic review and meta-analysis were conducted via an electronic search of the following databases: MEDLINE, EMBASE, and the Cochrane Central Register of Controlled Trials (CENTRAL). All papers comparing RA to either NS or CF for posterior spinal fusion in scoliosis were included. Fixed and random effects models of analysis were utilised based on analysis heterogeneity.

**Results:**

10 observational studies were included in total. RA had significantly greater odds of accurate pedicle screw placement relative to both NS (OR = 2.02, CI = 1.52–2.67, p < 0.00001) and CF (OR = 3.06, CI = 1.79–5.23, p < 0.00001). The downside of RA was the significantly greater operation duration relative to NS (MD = 10.74, CI = 3.52–17.97, p = 0.004) and CF (MD = 40.27, CI = 20.90, p < 0.0001). Perioperative outcomes including estimated blood loss, radiation exposure, length of hospital stay, cobb angle correction rate, postoperative SRS score, VAS pain score, JOA score, as well as rates of neurological injury and revision surgery, were comparable between the groups (p > 0.05).

**Conclusion:**

RA offers significantly greater pedicle screw placement accuracy relative to NS and CF, however, surgery can take longer. In terms of perioperative outcomes, all three techniques are comparable.

## Introduction

Scoliosis is a three-dimensional deformity marked by coronal and sagittal curvature of the spine with varying degrees of spinal rotation [1]. In severe or progressive cases, surgical correction for spinal fusion is necessary [2]. One of the key components of surgical correction is the placement of pedicle screws,this allows for 3-column fixation and deformity correction manoeuvres. Scoliosis is associated with three-dimensional anatomical complexity including vertebral rotation and small dysplastic pedicles in the curve concavity [3]. The deformity encountered in scoliosis makes pedicle screw placement technically challenging which elevates the risk of screw misplacement as well as potential complications such as neurological injury, visceral injury, or revision surgery [4].

At present, the primary approach for pedicle screw implantation is the conventional free-hand technique (CF) [5]. Despite careful pedicle tapping for accurate determination of screw pathway, pedicle screws may be inaccurately placed due to atypical anatomical complexity including axial rotation as well as pedicle calibre [1]. Screw misplacement continues to be the predominant form of instrument-related complication in scoliosis surgery, posing a significant concern for both patients and spinal deformity surgeons [5].

Various techniques have been developed to assist accurate pedicle screw insertion, including spinal navigation systems (NS) and robot-assisted (RA) technologies [6]. Navigation involves computerized image processing visualization system that provides crucial intraoperative assistance for screw placement. This is commonly in the form of 3D fluoroscopy or intraoperative computed tomography (CT) scan to monitor the patient’s anatomical position along with infrared stereoscopic positioning technology to track the surgical instrument location, ensuring high precision [7].

In recent years, there has been extensive interest and research relating to the role of robot-assisted (RA) technology in the spine surgery [8]. The goal of RA surgery is to address manual surgeon errors, commonly seen in more conventional techniques, and allowing better surgical planning [9]. This primarily consists of a robotic arm, an optical tracking system, and a surgical navigation system,together these components work to establish a clear surgical plan with precise pedicle screw trajectories and real-time monitoring. The challenge with this system is the absence of tactile feedback during screw placement [10].

The objective of this study is to evaluate and contrast the safety and efficacy of distinct pedicle screw insertion methods including CF techniques, NS, and RA surgery. More specifically RA surgery will be compared to both NS and CF techniques. To the best of our knowledge, this is the first systematic review and meta-analysis to consider these two comparisons together, specifically within the scoliosis population.

## Methods

A systematic review and meta-analysis were conducted as per the Preferred Reporting Items for Systematic Reviews and Meta-Analyses (PRISMA) guidelines [11].

### Eligibility criteria

The aim was to assess and compare RA surgery to NS and CF surgery. All observational studies directly comparing RA to either one of these groups were included. Scoliosis deformity was defined as greater than 10 degrees measurement of main thoracic coronal cobb angle**.**

### Primary outcomes

The primary outcome was acceptable pedicle screw placement as per the Gertzbein-Robbins grading system [12]. This classification system classifies pedicle screw position into 5 grades (A-E) based on postoperative CT. A grade A screw has no breach of the pedicle cortex. A grade B has a breach < 2 mm. A grade C has a breach between ≥ 2 but < 4 mm. A grade D has a breach ≥ 4 mm. A Grade E has a breach of ≥ 6 mm. Pedicle screws classified as either A or B are considered clinically acceptable.

### Secondary outcomes

Secondary outcomes included: radiation exposure in (mSV), operation duration in minutes (mins) and estimated blood loss (EBL) in millilitres (mL). length of hospital stay (LOS), deformity correction rate (percentage change in cobb angles), Total Scoliosis Research Society SRS-Score, postoperative Visual Analogue Score (VAS) for pain and postoperative Japanese Orthopaedic Association (JOA) score.

### Literature search strategy

A search of electronic databases of the following databases was performed by two authors independently: MEDLINE, EMBASE, and the Cochrane Central Register of Controlled Trials (CENTRAL). The last search was run on the 14th of November 2023. In addition, World Health Organization International Clinical Trials Registry (http://apps.who.int/trialsearch/), ClinicalTrials.gov (http://clinical-trials.gov/), and ISRCTN Register (http://www.isrctn.com/) were searched for any ongoing or unpublished studies. No language restrictions were applied in our search strategies. The search terminologies included ‘robot’, ‘deformity’, ‘scoliosis’, ‘navigation’, ‘CT’, ‘freehand, ‘fluoroscopy’, ‘O-arm’, ‘C-arm’.

### Selection of studies

Two authors independently assessed the titles and abstracts of the identified studies. Full-texts of relevant studies were obtained and those that met our eligibility criteria were chosen. Any discrepancies in study selection were resolved via group discussion between the authors.

### Data extraction and management

As per the Cochrane’s data collection form for intervention reviews, a spreadsheet was pilot-tested in randomly selected articles and was adjusted accordingly. This sheet included study-related data, baseline demographics of the included patients, as well as primary and secondary outcome data. Our data extraction spreadsheet included study-related data (first author, year of publication, country of origin of the corresponding author, journal in which the study was published, study design, study size, clinical condition of the study participants, type of intervention, and comparison), baseline demographics of the included populations (age and gender) and primary and secondary outcome data. Two authors collected and recorded the findings and any disagreements were resolved through discussion.

### Data analysis

Review Manager 5.3 software was used for data analysis. The collected data was entered into the software by two independent authors. A fixed effects model was used for outcomes with a heterogeneity less than 50%. A random effects model was used for outcomes with heterogeneity over 50%. 95% Confidence Intervals (CIs) were used in the forest plots. For dichotomous outcomes, the Odds Ratio (OR) was calculated between the two groups whereas for continuous outcomes, the Mean Difference (MD) was used.

### Assessment of heterogeneity

Study heterogeneity was assessed using the Cochran Q test (χ^2^) and was quantified by calculating I^2^. It was interpreted as follows: 0 to 25% as low heterogeneity, 25 to 75% as moderate heterogeneity, and 75 to 100% as high heterogeneity.

### Quality assessment

Using the Cochrane collaboration tool, risk of bias was assessed in randomized studies. The quality of all non-randomized studies was assessed via the Newcastle Ottawa Scale; this involves a star system to analyse study selection, comparability and outcome [13].

### Sensitivity analysis

A sensitivity analysis was carried out looking at the role of individual studies on the result of the forest plot. This assesses for any skewing of the results by any one study. One study was excluded from the analysis at any one time to look at the impact of any one study on overall significance. Individual studies or those with a high risk of bias did not independently impact the significance of the data. This was supported by funnel plots analysis.

## Results

### Literature search results

**Fig. 1:**
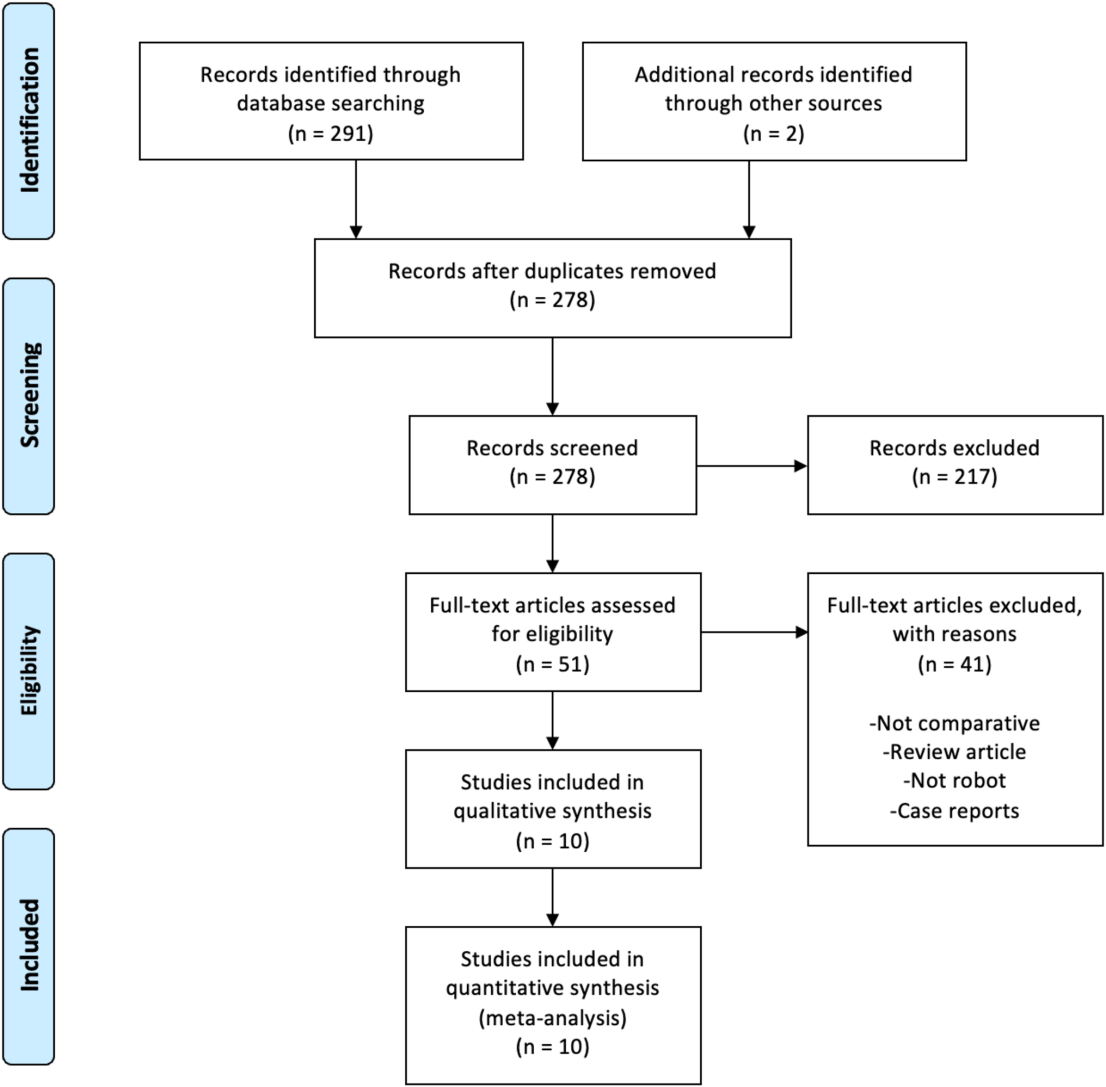
Prisma Flow Diagram. The PRISMA diagram representing the search and selection processes applied during the overview. PRISMA, Preferred Reporting Items for Systematic Reviews and Meta-Analyses [11]

The literature search identified 291 studies and after a thorough screening of the retrieved articles a total of 10 studies met the eligibility criteria (Fig. 1).

### Baseline characteristics

The baseline demographic data of included studies can be seen in Table 1.

**Table 1:** Baseline characteristics of included studies. RA vs NS vs CF

**Study author**	**Mean age in years (SD)**	**Gender (F:M)**	**Total of patients (**RA vs NS or CF)	**Type of intervention**	**Diagnosis of scoliosis**
Akazawa et al. 22	16.4 (2.7) vs 17.3 (2.6)	42:8	50 (18 vs 32)	Mazor X robot vs O-arm CT-navigation	AIS
Fan et al. 18	61.6 (9.1) vs 64 (7.7) vs 63.9 (8.4)	113:79	192 (109 vs 83)	Renaissance TM vs. O-arm CT navigation vs. fluoroscopy	ADS
Li et al. 23	14.9 (3.1) vs 15.3 (2.9) vs 15.4 (2.9)	83:23	106 (32 vs 34 vs 40)	TiRobot vs O-arm CT navigation vs CF	AIS, CON, NMD
Shuai Li et al. 23	13.2 (3.92) vs 14.6 (2.97)	27:33	60 (40 vs 20)	TiRobot vs O-arm CT navigation	AIS and CON
Chen et al. 20	69.8 (3.8) vs 69.3 (5.1)	60:37	97 (31 vs 66)	TiRobot VS CF	ADS
Haojie et al. 21	NA	NA	46 (22 vs 24)	TiRobot VS CF	AIS
Hou et al. 23	14.69 (1.93) vs 14.9 (2.01)	70:31	101 (45 vs 56)	Renaissance TM robot VS CF	AIS
Chao Li et al. 22	32.2 (22.8) vs. 29.1 (22.1)	103:41	144 (92 VS 52)	TiRobot VS CF	CON, AIS, NMD, ADS
Xiaoming et al. 23	NA	NA	40 (18 vs 22)	TiRobot vs CF	AIS
Linden et al. 22	15 (2.01) vs 15.3 (1.9)	46:14	60 (30 vs 30)	Mazor X robot + O-arm vs CF	AIS

*AIS* Adolescent idiopathic scoliosis; *ADS* Adult degenerative scoliosis; *CF* Conventional fluoroscopy assisted technique; *CON* Congenital scoliosis; *NMD* Neuromuscular scoliosis

Robot and navigation technology: Mazor X robot (Mazor X Stealth Edition, Medtronic Inc., Dublin, Ireland); Renaissance TM robot. TiRobot, TINAVI Medical Technologies Co., Ltd., Beijing, China). CT-navigation (Stealth Station S7, Medtronic Inc., Dublin, Ireland). (1–10)

## Primary outcome—RA vs. NS

### *Screw placement accuracy (% A* + *B accuracy)*

**Fig. 2:**
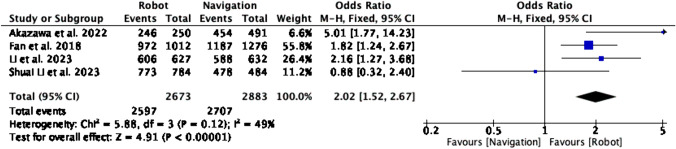
Forest plot for screw placement accuracy in RA vs NS

In Fig. 2, screw placement accuracy is compared between the RA and NS groups. From a total of 4 studies, a total of 5556 screws were placed. There were greater odds of placing pedicle screws in a clinically acceptable position in the RA group relative to the NS group (OR = 2.02, CI = 1.52–2.67, P < 0.00001). The level of heterogeneity was moderate (I^2^ = 49%, P = 0.12).

## Secondary outcomes for RA vs. NS

### Operation duration (minutes)

**Fig. 3:**
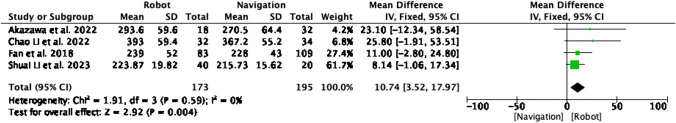
Forest plot of operation duration in RA vs NS

In Fig. 3, operation duration is compared between the RA and NS groups. From a total of 4 studies, 368 patients were enrolled. Operation durations were significantly greater in RA (MD = 10.74, CI = 3.52–17.97, P = 0.004). The level of heterogeneity was low (I^2^ = 0%, P = 0.59).

### Radiation exposure (mSV)

**Fig. 4:**
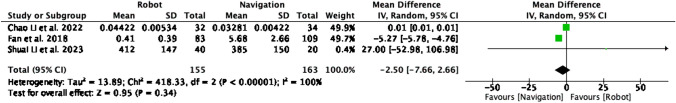
Forest plot of radiation exposure (mSV) in RA vs NS

Figure 4 reports radiation exposure in 3 studies with a total of 318 patients. No statistical significance was seen between the two groups (MD = –2.5, CI-7.66–2.66, P = 0.34) in terms of radiation exposure (mSV). Heterogeneity for this analysis was high (P = 100%, P < 0.00001).

### Estimated blood loss (milliliters)

**Fig. 5:**
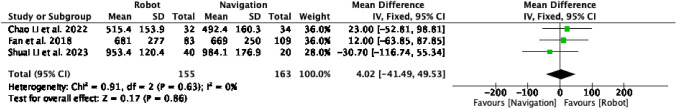
Forest plot of estimated blood loss (mL) in RA vs NS

Figure 5 reports EBL in 3 studies with a total of 318 patients with no statistically significant difference between the groups (MD = 4.02, CI = -41.49–49.53, P = 0.86). A low level of heterogeneity was present (I^2^ = 0%, P = 0.63).

### Length of hospital stay (LOS) in days

**Fig. 6:**
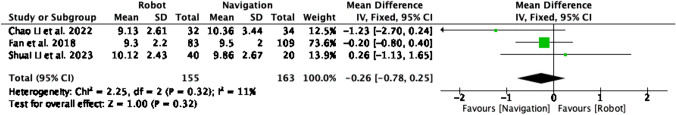
Forest plot for length of hospital stay postoperatively

In Fig. 6, LOS was reported in 3 studies enrolling a total of 318 patients with no statistically significant difference between the two groups (MD = –0.26, CI = –0.78–0.25, P = 0.32). A low level of heterogeneity was present (I^2^ = 11%, P = 0.32).

## Primary outcome for RA vs. CF

### *Screw placement accuracy (% A* + *B accuracy)*

**Fig. 7:**
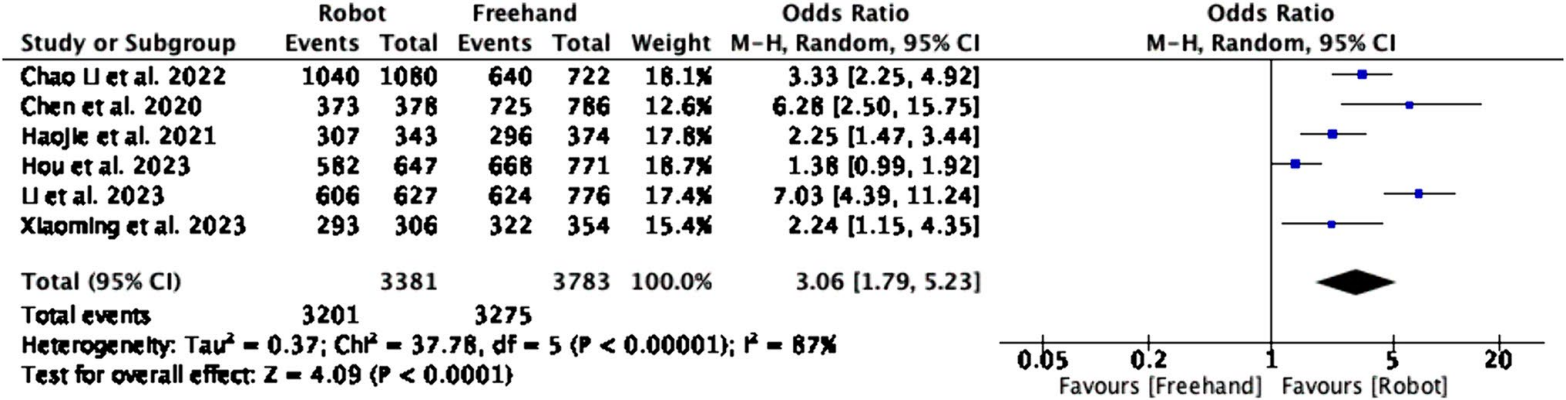
Forest plot for screw placement accuracy in RA vs CF

As seen in Fig. 7, screw placement accuracy was reported in 6 studies with a total of 7164 screws inserted. The RA group had significantly greater odds of placing screws with greater accuracy and in a clinically acceptable position (OR = 3.06, CI = 1.79–5.23, P < 0.00001). Study heterogeneity was high (I^2^ = 87%, P < 0.00001).

## Secondary outcomes for RA vs. CF

### Operation duration (minutes)

**Fig. 8:**
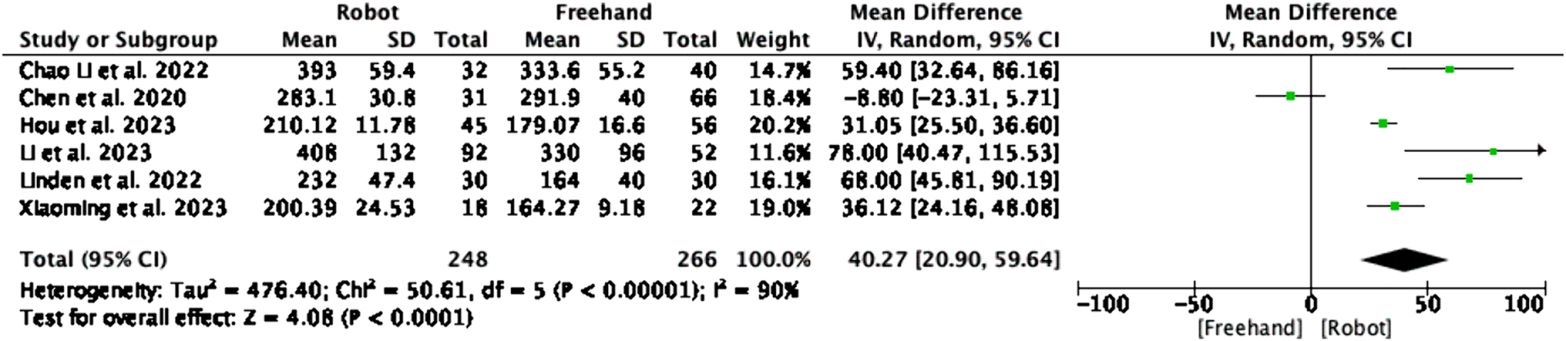
Forest plot of operation duration in RA vs CF

Figure 8 reports operation duration in 6 studies enrolling a total of 514 patients. The RA group had significantly greater operation durations than the CF group (MD = 40.27, CI = 20.90, P < 0.0001). Heterogeneity was high (I^2^ = 90%, P < 0.00001).

### Radiation exposure (mSV)

**Fig. 9:**
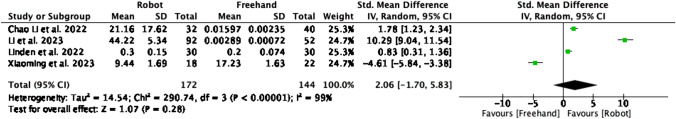
Forest plot of radiation exposure in RA vs CF

Radiation exposure was reported in 4 studies enrolling 316 patients (Fig. 9). No significance was noted between the groups (SMD = 2.06, CI = -1.7–5.83, P = 0.28). Heterogeneity was high (I^2^ = 98%, P < 0.00001).

### Estimated blood loss (milliliters)

**Fig. 10:**
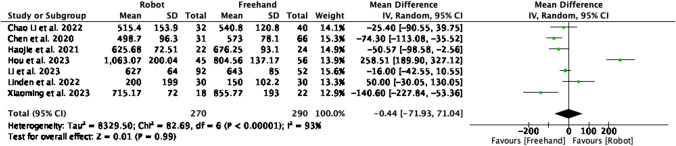
Forest plot of estimated blood loss

EBL was reported in 7 studies with a total of 560 patients (Fig. 10). No significance was noted between the two groups (MD = -0.44, CI = -71.93–71.04, P = 0.99). A high level of heterogeneity was found amongst the studies (I^2^ = 93%, P < 0.00001).

## LOS in days

**Fig. 11:**
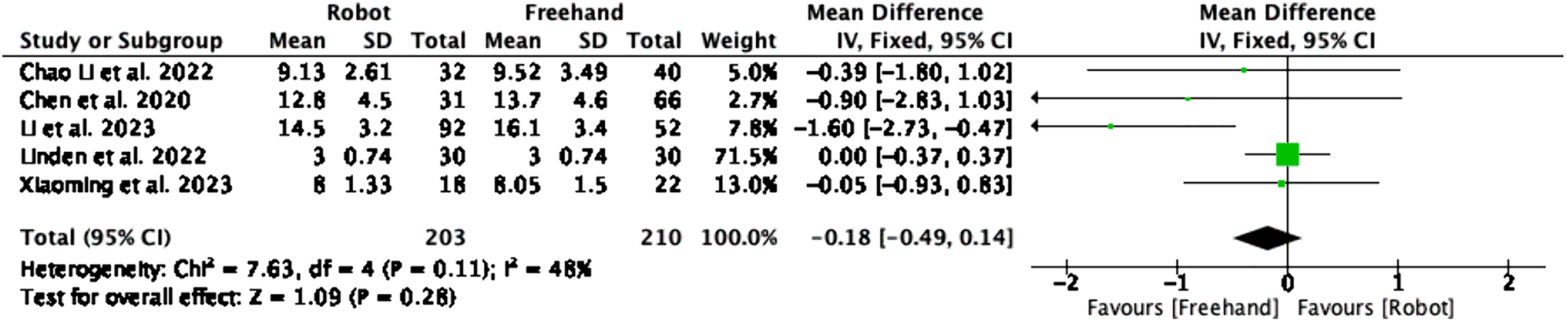
Forest plot of length of hospital stay

Length of hospital stay was reported in 5 studies enrolling a total of 413 patients (Fig. 11). There was no statistically significant difference between the groups (MD = -0.18, CI = -0.49–0.14, P = 0.28). A medium level of level of heterogeneity was found amongst the studies (I^2^ = 48%, P = 0.11).

### Cobb angle correction rate (%)

**Fig. 12:**
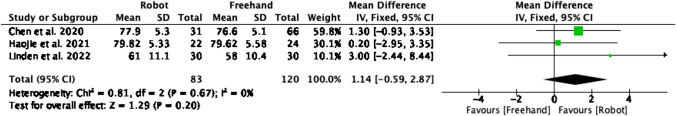
Forest plot of cobb angle correction rate

Cobb angle correction rate was reported in three studies enrolling a total of 203 patients (Fig. 12). Comparing the two groups, no statistically significant difference was seen (MD = 1.14, CI = -0.59–2.87, P = 0.20). A medium level of heterogeneity was present (I^2^ = 0%, P = 0.67).

## Total SRS-score

**Fig. 13:**
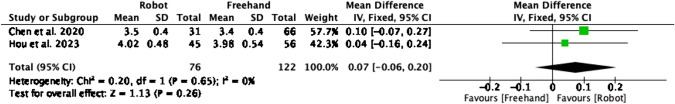
Forest plot of Total SRS score in RA vs CF

Total SRS score was reported in two studies enrolling a total of 198 patients (Fig. 13). Comparing the two groups, no statistically significant difference was seen (MD = 0.07, CI = -0.06–0.20, P = 0.26). A medium level of heterogeneity was present (I^2^ = 0%, P = 0.65).

### Postoperative VAS pain score

**Fig. 14:**
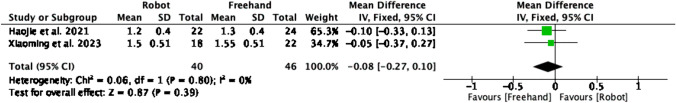
Forest plot of postoperative VAS pain score in RA vs CF

Postoperative pain VAS score was reported in two studies enrolling a total of 86 patients (Fig. 14). Comparing the two groups, no statistically significant difference was seen (MD = -0.08, CI = -0.27–0.10, P = 0.39). A low level of heterogeneity was present (I^2^ = 0%, P = 0.80).

### Postoperative JOA score

**Fig. 15:**
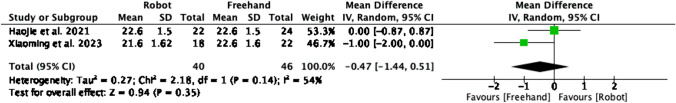
Forest Plot of postoperative JOA score in RA vs CF

Postoperative JOA score was reported in two studies enrolling a total of 86 patients (Fig. 15). Comparing the two groups, no statistically significant difference was seen (MD = -0.47, CI = -1.44–0.51, P = 0.35). A medium level of heterogeneity was present (I^2^ = 54%, P = 0.14).

## Complications

Table 2 represent the complications across different studies. RA shows superior outcomes in terms of screw placement accuracy compared to NS and CF. Additionally, NS shows superior screw placement accuracy relative to CF. No significant difference was seen in neurological complications or surgical revision rates. Revision surgery was mainly due to neurological or screw-related complications including loosening or malposition however reporting of this data is limited. Table 3

**Table 2:** Surgical complications

Study	Screw misplacement	Facet joint violation	Neurological injury	Revision surgery
Li et al. 23	3.3 vs 7.0 vs 19.6%	NA	RA 0 vs NS 0 vs CF 2	RA 0 vs NS 0 vs CF 1
Chen et al. 20	RA 1.3 vs CF 7.7%	NA	RA 0 vs CF 2	RA 0 vs CF 0
Chao Li et al. 22	RA 3.7 vs CF 11.4%	NA	RA 0 vs CF 2	RA 0 vs CF 1
Shuai Li et al. 23	RA 1.24 vs NS 12.4%	RA 4.07% vs NS 10.12	RA 0 vs NS 2	RA 0 vs NS 2
Fan et al. 18	RA 4 vs NS 7%	RA 1.1% vs NS 2.4%	RA 0 vs NS 1	RA 2 vs NS 4
Akazawa et al. 22	RA 1.6 vs NS 7.5%	NA	NA	NA
Haojie et al. 21	10.5 vs 20.9%	NA	NA	NA
Hou et al. 23	RA 9.3 vs CF 11.2%	NA	RA 0 vs CF 0	RA 1 vs CF 0
Xiaoming et al. 23	RA 4.2 vs CF 9%	NA	NA	NA
Linden et al. 22	NA	NA	NA	NA

**Table 3:** Quality assessment of nonrandomized studies using Newcastle–Ottawa classification

Study	Selection	Comparability	Outcome
Akazawa et al. 22	*****	**	**
Chen et al. 20	***	**	**
Fan et al. 18	****	**	**
Hou et al. 23	***	**	**
Li et al. 2023	***	**	**
Shuai Li et al. 23	***	*	**
Chao Li et al. 22	****	**	**
Linden et al. 22	***	**	**
Xiaoming et al. 23	***	*	**
Haoije et al. 21	***	*	**

### Quality assessment results

Overall, all studies were of high quality based on the Agency for healthcare and research quality (AHRQ) standards. Quality of the non-randomized studies was assessed utilising the Newcastle Ottawa Scale which uses a star system to analyse selection, comparability and outcome. All 10 nonrandomized studies demonstrated a high quality of patient selection with clear inclusion and exclusion criteria. Patients who underwent both RA and CF were obtained from the same database. Clear comparability was found in most studies with similar preoperative patient characteristics including age, BMI, type of scoliosis and cobb angles. Follow up duration was adequate, but not enough in some studies to assess postoperative outcome measures such as VAS and ODI.

### Discussion

Relative to both NS and CF techniques, RA surgery was superior in terms of pedicle screw placement accuracy with significantly greater odds of achieving clinically acceptable pedicle screw positioning. The downside with RS relative to both other groups was the significantly greater operation durations. What is important to note is that intraoperative and postoperative outcomes between the groups were all comparable including: EBL, radiation exposure, LOS, cobb angle correction rate, SRS-score, postoperative VAS pain score and postoperative JOA score.

The role of RA surgery in orthopaedics and spine surgery is still evolving but many studies have demonstrated associations with enhanced intraoperative and postoperative outcomes [24, 25]. A meta-analysis looking at RA knee arthroplasty showed better component positioning and alignment relative to conventional methods, however similar to our study, operation durations were significantly prolonged in the RA group [26]. Another meta-analysis looking at RA hip arthroplasty demonstrated greater implant accuracy and reduced limb length discrepancies. Despite these advantages, operation durations were also extended with no significant differences in complications and implant positioning [27]. Sun et al. performed a meta-analysis of 20 RCTs comparing RA to CF in spine surgery. The cohort consisted mainly of patients with traumatic fractures and degenerative changes. Similar to our results, they showed increase screw placement accuracy with RA with minimal clinical benefits [28].

Owing to anatomical complexity, small pedicle sizes and challenging vertebral rotation in scoliosis, the risk of misplacement and clinical complications is higher. Within spine surgery, the role of RA is primarily to improve the accuracy of pedicle screw insertion [29]. Screw placement accuracy is vital to avoid neurologic, vascular or visceral harm as well as the need for revision surgery [30]. With CF surgery, screw misplacement rates can range from 2 to 31% and is significantly dependent on surgical expertise [31]. Most commonly the Gertzbein-Robins scale is used which grades screw position from A to E, with screws being clinically acceptable if graded A or B [12]. A study by Zhang et al. showed significantly greater rates of clinically acceptable screws in RA (98.3%) relative to CF (93.6%) (p = 0.024). Compared to NS, RA also achieves higher screw placement accuracy although the difference is less than that for CF [32].

It would be useful to understand the role of RA and NS in aiding complex pedicle screw insertion, particularly at the concavity of the curve apex where pedicles are dysplastic. This was only assessed by Chao Li et al. 2023, who showed that concave sided pedicle screw misplacement was less in RA relative to NS and CF. The reported rates of lateral sided concave pedicle screw deviation were 1.4, 2.2 and 10.8% in RA, NS and CF respectively. On the medial concave side, RS and NS had no occurrence of pedicle screw misplacement whereas, CF had a reported rate of 3.9% [16].

Screw placement malposition may result in major complications such as CSF leak, nerve root irritation, vessel damage or even spinal cord injury [33]. However, despite the greater screw placement accuracy in RA relative to NS and CF, postoperative outcomes are comparable. This is widely supported across the literature where postoperative cobb angle correction and outcome measures such as VAS and ODI are similar between groups postoperatively [34–36]. Additionally, rates of neurological injury and surgical revision, are also similar between the groups [8, 15, 37]. This is mainly because neurological complications arise from deformity correction as oppose to pedicle screw placement [8].

Minimizing intraoperative radiation exposure is imperative, both for the surgical team and the patient. Although our meta-analysis did not show any difference in radiation exposure between the groups, a meta-analysis for studies in spine surgery in general showed reduced radiation exposure with RA relative to CF [34]. Khan et al. who compared radiation doses in RA to NS showed no significant difference between the two in radiation exposure [35].

Theoretically, RA surgery should reduce cognitive and technical load and thus make surgery both faster and more accurate however, real-world data remains controversial [33]. A lower operation duration is important as it is associated with a reduced risk of surgical site infection and shorter postoperative hospital stay. Similar to our study, a meta-analysis of RCTs for spine surgery in general shows significantly longer operation durations in RA surgery however, controversy still exists within the literature [34, 35]. It is important to consider impact of the learning curve associated with RA, expertise of the surgical team including radiographers, time for registration, as well as the type of robot used [38]. A study comparing operation durations throughout the learning curve showed reduced operation durations for posterior spinal fusion after 17 to 18 cases [39]. The cost of RA systems as well as any associated training required must not be neglected when deciding between techniques, especially since evidence for improved clinical outcomes with RA is still limited [40].

RA surgery has become routine practice in many surgical specialties; with this comes growing challenges and future considerations. Urologists have successfully managed to adopt the da-Vinci robot into routine care, since FDA approval in 2001 and much can be learnt from this process within orthopaedics to improve implementation, tackle the learning curve and ensure patient safety [41]. It is important to acknowledge the imperfection of robotic systems and understand the technical difficulties surgeons may face including equipment failure and other robot-related complications [42]. System failures should not compromise patient safety and overall care, and surgeons should be able to continue the procedure should they fail [43].

The dilemma with this is that if RA becomes routine practice, then how will future generations be trained on traditional techniques, and should robotic surgery be part of the standardized curriculum [44]. Intraoperative neuromonitoring, which is used to assess neurological injury in spine surgery, is a valuable tool that has been implemented as routine practice in many places [45]. When neuromonitoring alerts occur, checklists are commonly used to assess the patient and ensure that the team takes systematic and standardized actions to maintain patient safety [46]. Similarly, to address robotic system malfunction, it would be useful to develop checklists and standardized work processes to reduce variability, improve team work and patient safety [43]. Detailed reporting of major robotic complications within the literature is necessary to allow us to tackle challenges, standardize workflow and improve care. Considering the limited evidence of clinical improvement with robotics in spine deformity, as well as the implementation challenges, it is important that the cost–benefit analysis is carefully assessed.

To our knowledge, this study is the first systematic review to assess the role of robotics in scoliosis specifically. Moreover, it is the first systematic review comparing RA to both NS and CF. With a total 14 forest plots, this review assesses a wide range of operative and postoperative outcomes. Despite these strengths, our study is not without limitations. This study is based on a total of 10 studies only most of which are retrospective in nature. Study heterogeneity exists, particularly with regards to surgical technique and type of robot used. It would also be useful for future studies to assess the cause of revision surgery and neurological complications in RA and NS as data from current studies is limited. This paper supports previous claims that RA and NS are superior to CF in accuracy but fails to show significant clinical benefits [33]. Moving forwards, it is important to consider performing larger prospective trials assessing the role of robotics in scoliosis correction, as well as cost and training repercussions [33].

## Conclusion

In scoliosis, RA surgery offers greater pedicle screw insertion accuracy than NS and CF however, RA operation durations are significantly longer. Intraoperative and postoperative outcomes are comparable between the groups. Larger trials looking at RA in scoliosis correction are needed to help clarify the relationship between these technologies, especially with regards to scoliosis. It is important to acknowledge the pros and cons of RA surgery and consider the role of this technology in future practice and surgical training.

## Abbreviations

CF: Conventional fluoroscopy
NS: Navigation system
RA: Robot assisted
RCT: Randomized control trial
CI: Confidence interval
OR: Odds ratio
MD: Mean difference
SMD: Standardized mean difference

## Declarations

### Data availability

The datasets generated and analysed during the current study are available from the corresponding author on reasonable request.

## References

[1] Janicki JA, Alman B (2007) Scoliosis: review of diagnosis and treatment (in Eng). Paediatr Child Health 12(9):771–776. 10.1093/pch/12.9.771 19030463 10.1093/pch/12.9.771 PMC2532872

[2] Aebi M (2005) The adult scoliosis (in Eng). Eur Spine J 14(10):925–948. 10.1007/s00586-005-1053-9 16328223 10.1007/s00586-005-1053-9

[3] Harfouch EB, Bunyan RF, Faraidy MA, Alnemari HH, Bashir S (2022) Ponte osteotomies increase risk of intraoperative neuromonitoring alerts in adolescent idiopathic scoliosis surgery (in Eng). Surg Neurol Int 13:154. 10.25259/sni_67_2022 35509562 10.25259/sni_67_2022 PMC9062905

[4] Gupta MC et al (2023) Intraoperative neuromonitoring predicts postoperative deficits in severe pediatric spinal deformity patients (in Eng). Spine Deform 12:109–118. 10.1007/s43390-023-00745-3 37555880 10.1007/s43390-023-00745-3

[5] Kim YJ, Lenke LG, Bridwell KH, Cho YS, Riew KD (2004) Free hand pedicle screw placement in the thoracic spine: is it safe? Spine (Phila Pa 1976) 29(3):333–342. 10.1097/01.brs.0000109983.12113.9b 14752359 10.1097/01.brs.0000109983.12113.9b

[6] Huang M, Tetreault TA, Vaishnav A, York PJ, Staub BN (2021) The current state of navigation in robotic spine surgery (in Eng). Ann Transl Med 9(1):86. 10.21037/atm-2020-ioi-07 33553379 10.21037/atm-2020-ioi-07 PMC7859750

[7] Sembrano JN, Polly DW Jr, Ledonio CG, Santos ER (2012) Intraoperative 3-dimensional imaging (O-arm) for assessment of pedicle screw position: Does it prevent unacceptable screw placement? (in Eng). Int J Spine Surg 6:49–54. 10.1016/j.ijsp.2011.11.002 25694871 10.1016/j.ijsp.2011.11.002 PMC4300877

[8] Matur AV, Palmisciano P, Duah HO, Chilakapati SS, Cheng JS, Adogwa O (2023) Robotic and navigated pedicle screws are safer and more accurate than fluoroscopic freehand screws: a systematic review and meta-analysis (in Eng). Spine J 23(2):197–208. 10.1016/j.spinee.2022.10.006 36273761 10.1016/j.spinee.2022.10.006

[9] Lieberman IH, Kisinde S, Hesselbacher S (2020) Robotic-assisted pedicle ecrew placement during spine surgery. JBJS Essent Surg Tech 10(2):e0020. 10.2106/jbjs.St.19.00020 32944411 10.2106/jbjs.St.19.00020 PMC7478327

[10] Hu X, Ohnmeiss DD, Lieberman IH (2013) Robotic-assisted pedicle screw placement: lessons learned from the first 102 patients (in Eng). Eur Spine J 22(3):661–666. 10.1007/s00586-012-2499-1 22975723 10.1007/s00586-012-2499-1 PMC3585630

[11] Moher D, Liberati A, Tetzlaff J, Altman DG (2009) Preferred reporting items for systematic reviews and meta-analyses: the PRISMA statement (in Eng). PLoS Med 6(7):e1000097. 10.1371/journal.pmed.1000097 19621072 10.1371/journal.pmed.1000097 PMC2707599

[12] Gertzbein SD, Robbins SE (1990) Accuracy of pedicular screw placement In vivo. Spine 15(1):11–14 2326693 10.1097/00007632-199001000-00004

[13] S. B. Wells GA, O‘Connell D, Peterson J, Welch V, Losos M, et al. . "The newcastle-ottawa scale (NOS) for assessing the quality of nonrandomized studies in meta-analyses " www.ohri.ca/programs/clinical_epidemiology/oxford.htm (accessed 10th Aug 2023).

[14] Akazawa T et al (2023) Comparison of radiographic and patient-reported outcomes after surgery in adolescent idiopathic scoliosis between robotics and navigation: an analysis using propensity score matching (in Eng). Cureus 15(11):e49061. 10.7759/cureus.49061 38116336 10.7759/cureus.49061 PMC10728579

[15] Fan Y, Peng Du J, Liu JJ, Zhang JN, Liu SC, Hao DJ (2018) Radiological and clinical differences among three assisted technologies in pedicle screw fixation of adult degenerative scoliosis (in Eng). Sci Rep 8(1):890. 10.1038/s41598-017-19054-7 29343756 10.1038/s41598-017-19054-7 PMC5772356

[16] Li C et al (2023) Safety and accuracy of cannulated pedicle screw placement in scoliosis surgery: a comparison of robotic-navigation, O-arm-based navigation, and freehand techniques (in Eng). Eur Spine J 32(9):3094–3104. 10.1007/s00586-023-07710-8 37273031 10.1007/s00586-023-07710-8

[17] Li S et al (2023) Comparison of surgical efficacy between O-arm combined with CT 3D real-time navigation system and Tinavi robot-assisted treatment of adolescent congenital scoliosis (in Eng). Am J Transl Res 15(5):3254–3266 37303634 PMC10250986

[18] Chen X et al (2020) Robot-assisted orthopedic surgery in the treatment of adult degenerative scoliosis: a preliminary clinical report (in Eng). J Orthop Surg Res 15(1):282. 10.1186/s13018-020-01796-2 32711566 10.1186/s13018-020-01796-2 PMC7382042

[19] Chen H, Zhu X, Dong L, Liu T (2021) [Study on robot-assisted pedicle screw implantation in adolescent idiopathic scoliosis surgery] (in Chi). Zhongguo Xiu Fu Chong Jian Wai Ke Za Zhi 35(11):1457–1462. 10.7507/1002-1892.202106072 34779173 10.7507/1002-1892.202106072 PMC8586781

[20] Hou C et al (2022) Comparison of robot versus fluoroscopy-assisted pedicle screw instrumentation in adolescent idiopathic scoliosis surgery: A retrospective study (in Eng). Front Surg 9:1085580. 10.3389/fsurg.2022.1085580 36756658 10.3389/fsurg.2022.1085580 PMC9899830

[21] C. Li *et al.*, "Comparison of the Accuracy of Pedicle Screw Placement Using a Fluoroscopy-Assisted Free-Hand Technique with Robotic-Assisted Navigation Using an O-Arm or 3D C-Arm in Scoliosis Surgery," *Global Spine Journal,* vol. 0, no. 0, p. 21925682221143076, doi: 10.1177/21925682221143076. 10.1177/21925682221143076 PMC11289529 36455162

[22] Xin Xiaoming GM, Fan Z, Fei C, Junchao F, Wenyuan L (2023) Application of orthopedic robot-assisted screw placement in the correction of adolescent idiopathic scoliosis. Chinese Journal of Tissue Engineering Research 27(36):5790–5794. 10.12307/2023.775 10.12307/2023.775

[23] Linden GS, Ghessese S, Cook D, Hedequist DJ (2022) Pedicle screw placement in adolescent idiopathic scoliosis: a comparison between robotics coupled with navigation versus the freehand technique. Sensors (Basel) 22(14):5204. 10.3390/s22145204 35890882 10.3390/s22145204 PMC9316760

[24] Al-Naseem AO, Gonnah AR, Al-Ali H, Al-Naseem AO, Siddique I (2022) Robot-assisted versus conventional freehand fluoroscopy-guided percutaneous screw fixation in femoral neck fractures: a systematic review and meta-analysis (in Eng). Cureus 14(4):e24258. 10.7759/cureus.24258 35607578 10.7759/cureus.24258 PMC9123337

[25] Al-Naseem A, Sallam A, Gonnah A, Masoud O, Abd-El-Barr MM, Aleem IS (2023) Robot-assisted versus conventional percutaneous sacroiliac screw fixation for posterior pelvic ring injuries: a systematic review and meta-analysis (in Eng). Eur J Orthop Surg Traumatol 33(1):9–20. 10.1007/s00590-021-03167-x 34842991 10.1007/s00590-021-03167-x

[26] Kort N, Stirling P, Pilot P, Müller JH (2022) Robot-assisted knee arthroplasty improves component positioning and alignment, but results are inconclusive on whether it improves clinical scores or reduces complications and revisions: a systematic overview of meta-analyses (in Eng). Knee Surg Sports Traumatol Arthrosc 30(8):2639–2653. 10.1007/s00167-021-06472-4 33666686 10.1007/s00167-021-06472-4

[27] Kumar V, Patel S, Baburaj V, Rajnish RK, Aggarwal S (2023) Does robotic-assisted surgery improve outcomes of total hip arthroplasty compared to manual technique? A systematic review and meta-analysis (in Eng). Postgrad Med J 99(1171):375–383. 10.1136/postgradmedj-2021-141135 37294729 10.1136/postgradmedj-2021-141135

[28] Sun WX et al (2023) Clinical efficacy of robotic spine surgery: an updated systematic review of 20 randomized controlled trials (in Eng). EFORT Open Rev 8(11):841–853. 10.1530/eor-23-0125 37909700 10.1530/eor-23-0125 PMC10646522

[29] Schizas C, Thein E, Kwiatkowski B, Kulik G (2012) Pedicle screw insertion: robotic assistance versus conventional C-arm fluoroscopy (in Eng). Acta Orthop Belg 78(2):240–245 22696996

[30] Modi HN, Suh SW, Hong JY, Yang JH (2010) Accuracy of thoracic pedicle screw using ideal pedicle entry point in severe scoliosis (in Eng). Clin Orthop Relat Res 468(7):1830–1837. 10.1007/s11999-010-1280-1 20182830 10.1007/s11999-010-1280-1 PMC2882019

[31] Galetta MS, Leider JD, Divi SN, Goyal DKC, Schroeder GD (2019) Robotics in spinal surgery (in Eng). Ann Transl Med 7(Suppl 5):S165. 10.21037/atm.2019.07.93 31624731 10.21037/atm.2019.07.93 PMC6778279

[32] Shafi KA et al (2022) Does robot-assisted navigation influence pedicle screw selection and accuracy in minimally invasive spine surgery? (in Eng). Neurosurg Focus 52(1):E4. 10.3171/2021.10.Focus21526 34973674 10.3171/2021.10.Focus21526

[33] Sielatycki J, Mitchell K, Leung E, Lehman R (2021) State of the art review of new technologies in spine deformity surgery–robotics and navigation. Spine Deform 10(1):5–17. 10.1007/s43390-021-00403-6 34487345 10.1007/s43390-021-00403-6 PMC8741671

[34] Tarawneh AM, Salem KM (2021) A Systematic review and Meta-analysis of randomized controlled trials comparing the accuracy and clinical outcome of pedicle screw placement using robot-assisted technology and conventional freehand technique (in Eng). Global Spine J 11(4):575–586. 10.1177/2192568220927713 32677515 10.1177/2192568220927713 PMC8119930

[35] Khan A et al (2020) Comparing cortical bone trajectories for pedicle screw insertion using robotic guidance and three-dimensional computed tomography navigation (in Eng). World Neurosurg 141:e625–e632. 10.1016/j.wneu.2020.05.257 32522651 10.1016/j.wneu.2020.05.257

[36] Lin MC, Liu HW, Su YK, Lo WL, Lin CM (2022) Robot-guided versus freehand fluoroscopy-guided minimally invasive transforaminal lumbar interbody fusion: a single-institution, observational, case-control study (in Eng). Neurosurg Focus 52(1):E9. 10.3171/2021.10.Focus21514 34973679 10.3171/2021.10.Focus21514

[37] Zhang JN, Fan Y, He X, Liu TJ, Hao DJ (2021) Comparison of robot-assisted and freehand pedicle screw placement for lumbar revision surgery (in Eng). Int Orthop 45(6):1531–1538. 10.1007/s00264-020-04825-1 32989559 10.1007/s00264-020-04825-1

[38] Ong V et al (2022) A comparison of spinal robotic systems and pedicle screw accuracy rates: review of literature and meta-analysis (in Eng). Asian J Neurosurg 17(4):547–556. 10.1055/s-0042-1757628 36570749 10.1055/s-0042-1757628 PMC9771638

[39] Yu J, Zhang Q, Fan MX, Han XG, Liu B, Tian W (2021) Learning curves of robot-assisted pedicle screw fixations based on the cumulative sum test (in Eng). World J Clin Cases 9(33):10134–10142. 10.12998/wjcc.v9.i33.10134 34904083 10.12998/wjcc.v9.i33.10134 PMC8638049

[40] Menger RP, Savardekar AR, Farokhi F, Sin A (2018) A cost-effectiveness analysis of the integration of robotic spine technology in spine surgery (in Eng). Neurospine 15(3):216–224. 10.14245/ns.1836082.041 30157583 10.14245/ns.1836082.041 PMC6226125

[41] Shah AA, Bandari J, Pelzman D, Davies BJ, Jacobs BL (2021) Diffusion and adoption of the surgical robot in urology (in Eng). Transl Androl Urol 10(5):2151–2157. 10.21037/tau.2019.11.33 34159097 10.21037/tau.2019.11.33 PMC8185660

[42] Jara RD, Guerrón AD, Portenier D (2020) Complications of robotic surgery. Surg Clin North Am 100(2):461–468. 10.1016/j.suc.2019.12.008 32169190 10.1016/j.suc.2019.12.008

[43] Sethi R, Bohl M, Vitale M (2019) State-of-the-art reviews: safety in complex spine surgery (in Eng). Spine Deform 7(5):657–668. 10.1016/j.jspd.2019.04.002 31495465 10.1016/j.jspd.2019.04.002

[44] Carpenter BT, Sundaram CP (2017) Training the next generation of surgeons in robotic surgery (in Eng). Robot Surg 4:39–44. 10.2147/rsrr.S70552 30697562 10.2147/rsrr.S70552 PMC6193443

[45] A. O. Al-Naseem *et al.*, "Does spinal cord type predict intraoperative neuro-monitoring alerts in scoliosis correction surgery? a systematic review and meta-analysis of operative and radiologic predictors," *Global Spine J,* vol. 0, no. 0, p. 21925682241237475, doi: 10.1177/21925682241237475. 10.1177/21925682241237475 PMC11418721 38428951

[46] Vitale MG et al (2014) Best practices in intraoperative neuromonitoring in spine deformity surgery: development of an intraoperative checklist to optimize response (in Eng). Spine Deform 2(5):333–339. 10.1016/j.jspd.2014.05.003 27927330 10.1016/j.jspd.2014.05.003

